# Oral Administration of *Flavonifractor plautii*, a Bacteria Increased With Green Tea Consumption, Promotes Recovery From Acute Colitis in Mice *via* Suppression of IL-17

**DOI:** 10.3389/fnut.2020.610946

**Published:** 2021-02-04

**Authors:** Ayane Mikami, Tasuku Ogita, Fu Namai, Suguru Shigemori, Takashi Sato, Takeshi Shimosato

**Affiliations:** Department of Biomolecular Innovation, Institute for Biomedical Sciences, Shinshu University, Nagano, Japan

**Keywords:** *Flavonifractor plautii*, green tea, inflammatory bowel disease, IL-17, lipoteichoic acid

## Abstract

*Flavonifractor plautii* (FP) has been reported to participate in the metabolism of catechins in the human gut. However, there is limited information on the immune regulatory effects of this bacterium. We confirmed that the administration of green tea increases the abundance of FP in the gut microbiota and investigated the effect of FP in a mouse colitis model. Mice were orally administered FP for 10 consecutive days; colonic inflammation was evaluated daily on the basis of stool consistency, gross rectal bleeding, and body weight. In the dextran sodium sulfate model, FP-exposed animals exhibited lower levels of inflammation and strong inhibition of interleukin (IL)-17 signaling. Moreover, lipoteichoic acid from FP was identified as the active component mediating IL-17 suppression. Thus, oral administration of FP appears to modulate gut inflammation and represents a viable and inexpensive oral microbial therapeutic.

## Introduction

Inflammatory bowel disease (IBD) is a generic term for chronic inflammatory disease in the gastrointestinal tract, typified by Crohn's disease and ulcerative colitis (1). IBD patients exhibit macrophage infiltration and excessive activation of tumor necrosis factor alpha (TNF-α) and interleukin (IL)-17 in local inflammation (2). This results in tissue injury, such as intestinal barrier disruption and ulceration (3), and the development of further hyperimmunity (4). Several studies reported that dysbiosis occurs in IBD patients, involving a reduction in Firmicutes, Bacteroidetes, and commensal bacterial diversity and higher populations of *Escherichia coli* and *Clostridium difficile* (5–8). In the last 30 years, the number of IBD patients has been rapidly increasing, mainly in Europe and the United States, and a similar trend in Japan has been observed (9, 10). A genetic predisposition and the involvement of environmental factors have been reported in the development of IBD (11, 12). However, in spite of the relatively homogeneous genetic background of the Japanese population, environmental factors seem to affect disease development more strongly than genetic predisposition, as evidenced by the rapid rise in the number of patients. In the 1970s, when the number of patients began to increase rapidly in Japan, Western food featuring high-fat and low-fiber content was pervasive (13), and consequent differences in diet and gut microbiota may have contributed to the occurrence of IBD. Moreover, the use of germ-free mice in investigating IBD suggests that alterations in the gut microbiota may be involved in IBD development (14). Intestinal fecal microbiota transplantation has been reported to successfully treat IBD patients (15). These reports suggest that alteration in the gut microbiota may be involved in the onset of IBD (16, 17).

In recent years, it has been reported that dysbiosis is involved not only in IBDs but also in other inflammatory diseases such as obesity and articular rheumatism (14, 18). In contrast, a healthy microbiota contributes to the host's health maintenance via the immune system (19). Interestingly, it has become evident that functional enterobacteria play a role in controlling the differentiation and maturation of immune cells in the host intestinal immune system (20). Segmented filamentous bacteria contribute to postnatal maturation of the intestinal immune system; specifically, T-helper-type 17 cell differentiation, immunoglobulin A (IgA) production, and intestinal barrier protection (20). These functions help protect against invading pathogens. For example, we reported that the common enterobacterium *Flavonifractor plautii* (FP) regulates inflammation in an obesity model (21). FP is a Gram-positive bacteria belonging to the Clostridiales order and is involved in catechin metabolism in the intestines. However, its functionality is poorly understood. In this study, we investigated the effect of the consumption of green tea, a common beverage in Japan, on the abundance of FP in the gut of mice. Following confirmation that the administration of green tea increases the abundance of FP in the gut microbiota, the physiological effects of FP were investigated in an IBD mouse model. Therefore, we investigated the *in vitro* and *in vivo* anti-inflammatory effect of FP, as well as the suppression of Th17 immune responses by FP, especially during the recovery phase of an IBD model.

## Materials and Methods

### Reagents

Cell culture reagents and supplies were purchased from Thermo Fisher Scientific, Inc. (Waltham, MA, USA, RRID:SCR_008452). Cells were cultured in complete Roswell Park Memorial Institute (RPMI) 1640 medium supplemented with 10% fetal bovine serum, 1% non-essential amino acids, and antibiotics (100 U/ml penicillin, 100 mg/ml streptomycin), 25 mM HEPES, 1.0 mM sodium pyruvate, and 0.0035% 2-mercaptoethanol. The green tea used in this study was commercially available in powdered form. All other chemicals were purchased from Nacalai Tesque (Kyoto, Japan, RRID:SCR_013519).

### Preparation of FP and Lipoteichoic Acid From FP

FP was purchased from the American Type Culture Collection (*Flavonifractor plautii* ATCC® 29863™, Manassas, VA, USA) and cultured as previously described (22). Briefly, FP was cultured in Gifu Anaerobic Medium (GAM) broth (Nissui Pharmaceutical Co., Ltd., Tokyo, Japan), and the cells were pelleted by centrifugation at 8,000 × *g*, 4°C for 5 min, and then washed with and resuspended in sterile water to yield a suspension at a density of 1 × 10^11^ colony-forming units (cfu)/mL. The resulting suspension was lyophilized, and the bacterial cells were stored at −80°C until used for the experiments. Lipoteichoic acid (LTA) was isolated as previously reported (23). For the morphological investigation of FP using scanning electron microscopy (SEM), FP was washed with phosphate-buffered saline (PBS), suspended in PBS, and then placed on a filter (Nanopercolator, JEOL, Tokyo, Japan). The liquid was suctioned using a 10 ml syringe, and the sample was fixed by immersion in 2.5% glutaraldehyde (TAAB Laboratories Equipment, Ltd., Berks, UK) for 1 h. After fixation, plasma membranes were fixed by lightly washing three times with 0.1 M cacodylate buffer and soaking for 1 h in 1% osmium. Following dehydration with ethanol, the samples were soaked in t-butyl alcohol, incubated twice at 52°C for 30 min, and stored at −80°C. Osmium coatings were carried out using the osmium coater Neoc-AN (Meiwafosis Co., Ltd., Tokyo, Japan). SEM images were obtained using a Miniscope TM4000PlusII (HITACHI High Technologies Corp., Tokyo, Japan) with an accelerating voltage of 15 kV.

### Mice

All animal experiments were conducted in accordance with the guidelines for animal experiments of Shinshu University. C57BL/6 mice (7 weeks, female) were obtained from Japan SLC (Hamamatsu, Japan) and housed under controlled conditions of temperature and light. A rodent diet (MF; Oriental Yeast Co., Ltd., Tokyo, Japan) and sterile water were provided *ad libitum*.

### Green Tea Intake in Mice

C57BL/6 mice (9 weeks, female) were acclimatized for 2 weeks before the start of the experiments. Mice were divided into two experimental groups (*n* = 7–8 each). There was no significant difference in body weight between the groups. Mice were supplied green tea or sterile water *ad libitum* for 10 days (*n* = 7–8). The green tea was prepared by dissolving powdered green tea at a concentration of 1% in boiling sterile water and incubating at 100°C for 5 min. The drinking bottles were changed daily. Water intake was measured every day. Body weight and food intake were measured every second day.

### Measurement of IL-17 in Feces

Fecal samples were prepared as previously reported (24). Feces were collected and stored −80°C until analysis. In brief, fecal samples were divided into 20 mg portions and homogenized in 100 μL of Halt Protease & Phosphatase Inhibitor Cocktail 100× and EDTA 100× (Thermo Fisher Scientific, Waltham, MA, USA) diluted 1:100. The mixture was centrifuged at 400 × *g* for 5 min at 4°C. The supernatant fluid was collected, and IL-17 levels were measured by ELISA (Mouse DuoSet ELISA kit, R&D Systems, Minneapolis, MN, USA) according to the manufacturer's instructions.

### Abundance of FP in Feces

DNA from fecal samples was extracted and purified using a QIAamp Fast DNA Stool Mini kit (Qiagen, Valencia, CA, USA) according to the manufacturer's protocol. FP abundance was measured by quantitative PCR (qPCR) as described previously (22, 25). Quantification of the target FP 16S ribosomal DNA (rDNA) levels was performed using the ΔΔCt method, with normalization to the total 16S rDNA content. External pure FP culture or feces-based FP standards were included in each run to construct a standard curve. The concentration (cfu/mg) of FP in each sample was calculated by comparing the Ct of the sample with that of the standard curve. Primers and cycling conditions were as described previously (22).

### DSS-Acute Colitis Model

C57BL/6 mice (9 weeks, female) were acclimatized for 2 weeks before the start of experiments. After acclimation, mice were divided into two experimental groups (*n* = 6 each, housed at 6 mice/cage). There was no significant difference in body weight between the groups. Day 0 of the untreated group (*n* = 6) was used as a baseline. Mice were exposed to 3% (w/w) dextran sodium sulfate (DSS) (M.W. 36,000–50,000 Da; MP Biomedicals, Aurora, OH, USA) in the drinking water *ad libitum* for 5 days. From day 5, they were given sterile water without DSS (26, 27). FP (10^8^ cfu/mouse) or PBS was administered via intragastric (i.g.) administration from day 0 to 9 (*n* = 12–19). The experimental schedule is shown in Figure 2C. During the experiment, body weight and Disease Activity Index (DAI) scores of the severity of colitis symptoms were measured daily (28). Body weight was expressed as a percentage of the initial (day 0) body weight. The DAI score is a combined score of body weight differences, diarrhea, and bloody stools and was evaluated according to a previously reported method (28). On days 0, 6, 8, and 10, mice were sacrificed, and measurements of colon length were obtained. Colon length was measured from the ileocecal valve to the end of the lower rectum. IL-17, IL-23, and IL-10 concentrations in colon homogenates were evaluated by ELISA (Mouse DuoSet ELISA kit, R&D Systems) according to the manufacturer's instructions. The cytokine levels per colon tissue are presented as pg/mg tissue protein in each sample.

### IL-17 Regulation Assay

Splenocytes (1.0 × 10^7^ cells/mL) from C57BL/6 mice (6 weeks, female) were stimulated by 50 ng/mL interleukin 6 (IL)-6 (R&D Systems) and 2.5 ng/mL transforming growth factor (TGF)-β1 (R&D Systems). At the same time, medium FP (10^8^ cfu/mL), heat-killed FP (10^8^ cfu/mL), FP-LTA (10 μg/well), and/or LTA from *Staphylococcus aureus* (SA-LTA; Sigma Aldrich, St. Louis, MO, USA) were added. Cultures were carried out for 72 h at 37°C in an atmosphere of 5% CO_2_. The culture supernatants were measured using the above-described ELISA kits.

### Statistical Analysis

All statistical analyses were performed using GraphPad Prism7 (GraphPad Software, Inc., La Jolla, CA, USA). *In vitro* data are presented as the mean ± standard deviation (SD). *In vivo* data are presented as the mean ± standard error of mean (SE). The statistical significance (*p* < 0.05) of differences was determined by a Student's *t*-test or a one-way analysis of variance (ANOVA).

## Results

### Effects of Green Tea Consumption on Fecal FP Abundance and IL-17 Production

**Figure 1:**
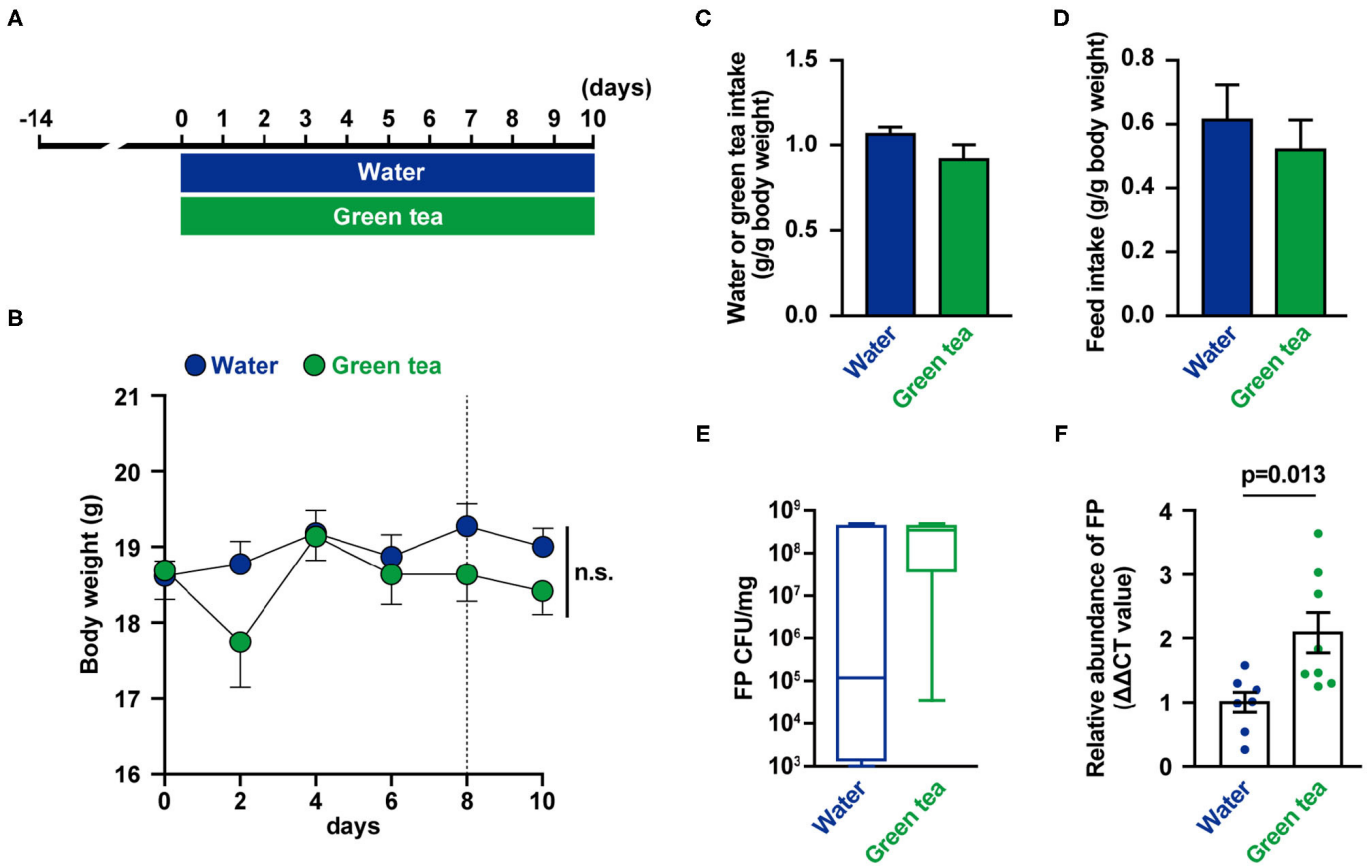
Effects of green tea intake on *Flavonifractor plautii* (FP) abundance and interleukin (IL)-17 production in mice. **(A)** Experimental schedule of green tea administration in mice. **(B)** Body weight was determined for each animal at each time point. **(C)** Water or green tea intake and **(D)** daily food intake data for the 10-day treatment period. **(E)** FP quantification (cfu/mg) and **(F)** relative FP 16S ribosomal DNA (rDNA) abundance in feces at day 8. Data are presented as the mean ± SE (*n* = 7–8). Statistical significance was assessed using a two-tailed non-paired Student's *t*-test. The data for the water and green tea groups are pooled from two independent experiments. n.s., not significant.

The mice were supplied distilled water or green tea every day for 10 days (Figure 1A). There were no differences in body weight, water intake, and food intake between groups (Figures 1B–D). On days 8 and 10, feces were collected, and FP abundance (Figures 1E,F) were measured. The relative abundance of FP in feces was higher in the green tea supplementation group on day 8 (Figure 1F). The fecal IL-17 levels were not significantly altered by green tea supplementation (data not shown).

### Morphological Observation of FP

**Figure 2:**
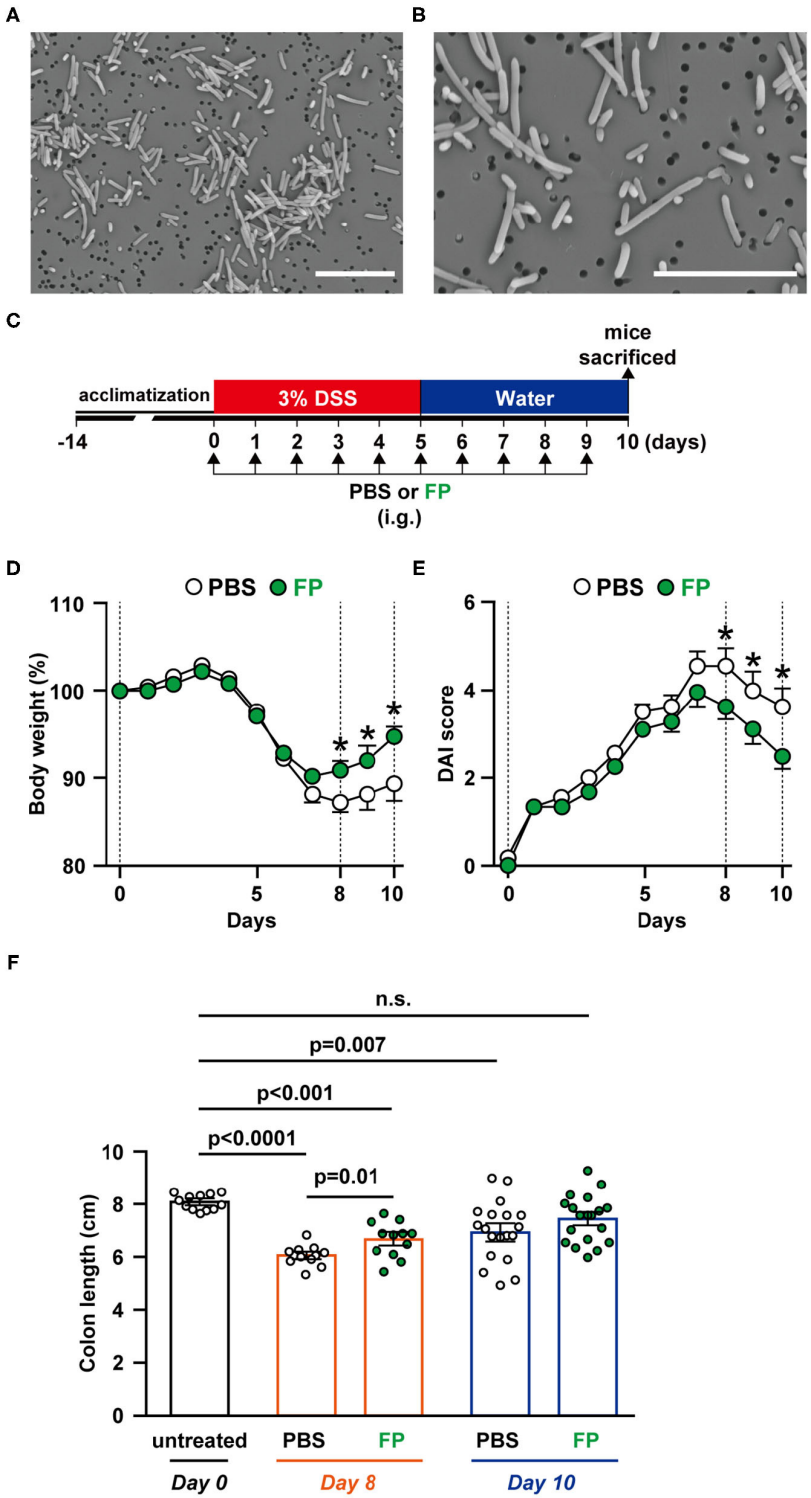
**(A,B)** Scanning electron microscopy (SEM) image of *Flavonifractor plautii* (FP). SEM photographs: **(A)** ×2,500 and **(B)** ×5,000 of FP. Scale bar = 10 μm. Effects of oral FP administration on dextran sodium sulfate (DSS)-induced colitis in mice. **(C)** Experimental schedule. **(D)** Body weight measurements and colitis symptoms as scored by **(E)** Disease Activity Index (DAI) or **(F)** colon length on days 0, 8, and 10. Colon length was measured from the base of the cecum to the anal constriction. *n* = 12–19 mice per group. Statistical significance was assessed using a two-tailed non-paired Student's *t-*test. The data for the phosphate-buffered saline (PBS) and FP groups are pooled from three independent experiments. n.s., not significant.

FP has been reported as a motile, Gram-positive, non-spore-forming bacillus (29). However, there have been no reports describing the morphology of FP. In this study, we observed FP using SEM; FP appeared as a bacillus that ranged in length from 1 to 8 μm. The surface of the cell wall was rough and lacked flagella (Figures 2A,B).

### FP Alleviates DSS-Induced Acute Colitis Symptoms

To evaluate the effects on FP on colitis symptoms, we conducted *in vivo* experiments using a DSS-induced acute colitis model (Figure 2C). Colons were collected on days 0, 6, 8, and 10 to observe temporal differences in internal colitis symptoms. The severity of colitis was evaluated using body weight (Figure 2D), DAI score (Figure 2E), and colon length (Figure 2F). Significant differences in body weight between the PBS and FP groups were observed on days 8–10 (Figure 2D). FP treatment significantly lowered DAI scores on days 8–10 (Figure 2E). Diarrhea and bloody stool scores were improved in mice administered FP on days 3 and 5, respectively (data not shown). Colon length of the FP group was longer compared to PBS on day 8 (Figure 2F). Significant improvement in the rate of body weight difference was observed in the FP group on days 8–10. In the FP group, a significant reversal of DSS-induced colon shortening was observed on day 8.

### FP Suppressed IL-17 Production in the Colon

**Figure 3:**
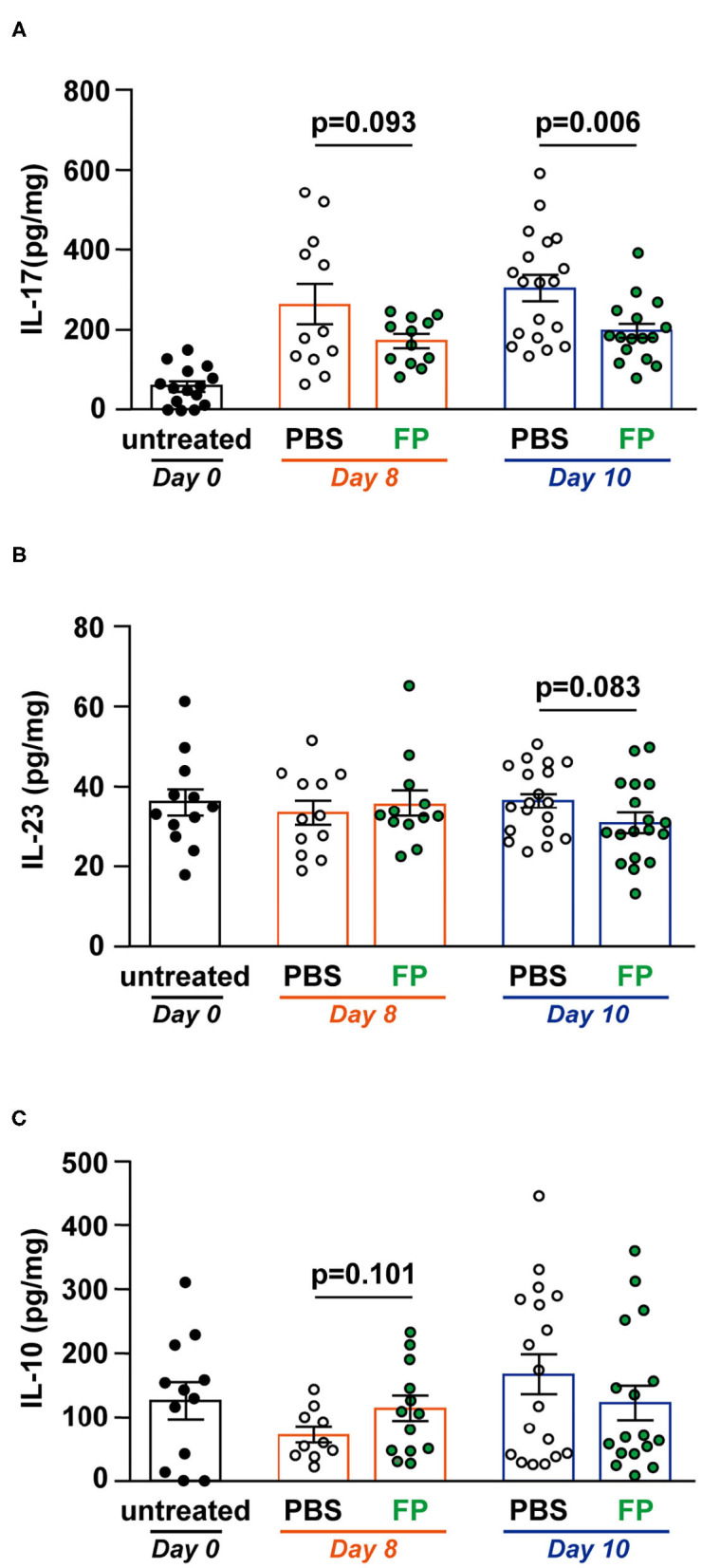
Effect of *Flavonifractor plautii* (FP) on interleukin (IL)-17, IL-10, and IL-23 levels in the colon tissue. **(A)** IL-17, **(B)** IL-23, or **(C)** IL-10 levels were evaluated in colon tissue homogenates collected on days 0, 8, and 10. Data are presented as the mean ± SE (*n* = 12–16). Statistical significance was assessed using a two-tailed non-paired Student's *t-*test. The data for the phosphate-buffered saline (PBS) and FP groups are pooled from three independent experiments.

Th17 cells massively infiltrate the intestine of IBD patients, where they mainly produce IL-17A. IL-17 signaling is triggered and amplified during the inflammatory process (30). IL-23 is a cytokine that maintains and proliferates Th17 cells in IBD (31). Therefore, we measured IL-17, IL-23, and IL-10 levels in colon tissues (Figures 3A–C). On day 10, IL-17 level was significantly lower in the colon of the FP group (*p* = 0.006) (Figure 3A). However, IL-23 and IL-10 production in colon tissues did not differ between groups (Figures 3B,C). Thus, the FP group exhibited lower IL-17 levels that were unrelated to IL-10 signaling. IL-23 is a Th17-related cytokine that activates gut inflammation, and its production is elevated in IBD patients (32). IL-10 is also known as a cytokine related to IL-17. These cytokines often show opposite expression patterns in IBD models; specifically, IL-17 levels are elevated in colitis mice (33). Consistent with this, there were no significant differences in IL-23 and IL-10 production associated with FP treatment on either day.

### FP-LTA Suppressed IL-17 Production

**Figure 4:**
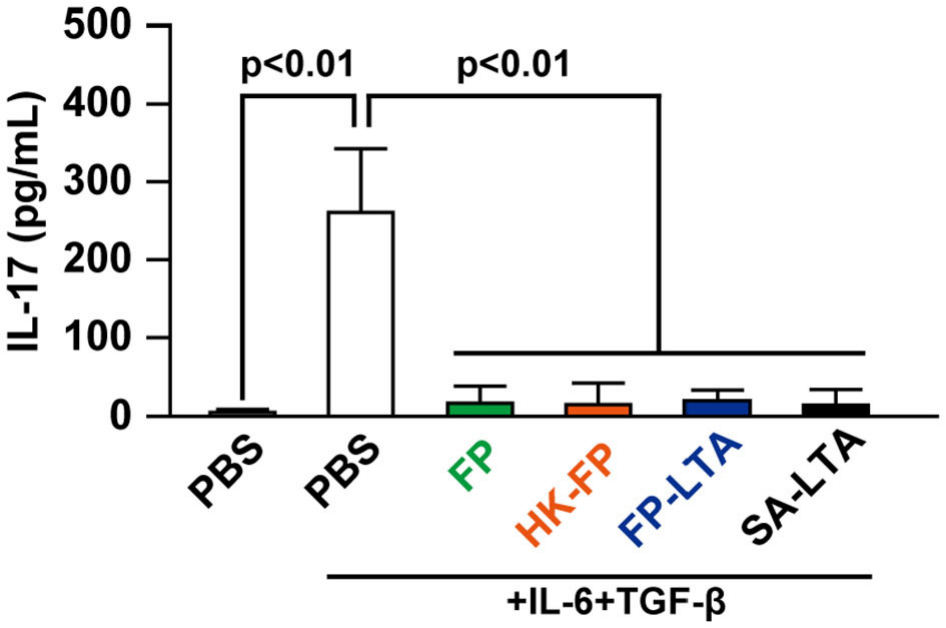
The effect of LTA on interleukin (IL)-17 levels in mouse splenocytes. IL-17 production in mouse splenocytes was determined in the presence and absence of IL-6/transforming growth factor (TGF)-β and various sources of LTA. Cellular supernatants were collected, and IL-17 levels were measured with ELISA. Values represent means, and error bars indicate the SD of three independent experiments. *p* < 0.01 by one-way analysis of variance followed by Tukey's multiple comparison test. At least three independent experiments were conducted in triplicate. HK, heat killed; SA, *Staphylococcus aureus*, LTA, lipoteichoic acid.

Son et al. (34) reported that bacterial LTA can reduce proliferation of effector CD4^+^ T cells. Therefore, we investigated the effect of FP-LTA on IL-17 suppression *in vitro*. Specifically, we focused on LTA, which is a major cell wall component of Gram-positive bacteria such as FP. Treatment of mouse splenocytes with the FP or SA-LTA, as well as live-FP and HK-FP, resulted in the inhibition of IL-17 production (Figure 4).

## Discussion

Although FP has been reported to be involved in the metabolism of catechins (35, 36), the impact of catechins on the relative abundance of FP has not been clarified. Here, we investigated the relationship between FP and catechins in mice administered green tea, a catechin-rich material. Catechins are antioxidants found in a variety of foods and constitute up to 42% of green tea by dry weight. The major catechins in green tea are (–)-epigallocatechin-3-gallate, (–)-epicatechin-3-gallate, (–)-epigallocatechin, and (–)-epicatechin (37). These catechins have been reported to exert a variety of beneficial effects including antioxidant (38), anti-inflammatory (39), antiobesity (40), and anticancer effects (37, 41). Green tea has also been reported to suppress IL-17 expression in rheumatoid arthritis, periodontitis, and IBD model mice (42). Interestingly, we showed that the abundance of FP was higher on day 8 with green tea drinking.

Next, we aimed to investigate the use of FP as part of the host's commensal gut bacteria in mitigating acute colitis. A DSS-induced colitis model was used to reproduce IBD symptoms and validate the efficacy of FP strains in reducing intestinal inflammation. Shortening of colon length is a symptom of IBD (43) and was improved on day 8 in mice fed FP. This suggests that FP relieves DSS-induced colitis symptoms. Recovery of these symptoms was not observed until days 6 and 7. Body weight and DAI scores did not differ between groups on day 6. Therefore, FP may promote recovery rather than suppress worsening of colitis symptoms. To investigate the responsible mechanisms, we focused on Th17. Th17 cells produce IL-17 and are the main immune cell involved in mucosal immunity and act to defend against extracellular bacterial and fungal infections. Overexpression of IL-17 has been reported to be associated with IBD pathogenesis and is abundantly found in inflamed IBD gut mucosa (44–47). We observed that IL-17 production in the colon tissue was lower in the FP group on days 6 and 10, indicating that FP alleviates mucosal damage by suppressing the overexpression of IL-17.

In the *in vitro* experiment, FP suppressed the production of IL-17. When splenocytes are stimulated with IL-6 and TGF-β, the main source of IL-17 is Th17 (IL-17^+^ and CD4^+^ T cells) (48). This result suggests that FP suppressed IL-17 production from Th17 cells. In addition, LTA preparations derived from FP (FP-LTA) also induced a decline in IL-17 production. LTA is a cell wall component of Gram-positive bacteria, and administration of LTA-deficient *Lactobacillus acidophilus* to mice decreased inflammatory signals (49). In contrast, LTA also exhibits anti-inflammatory effects by suppressing neutrophil migration (50). Since previous studies have not reported that LTA suppresses IL-17 expression, this result may be specific to bacterial-LTA.

FP is reported to be a flavonoid-degrading bacteria that is present in all mammals and has the ability to metabolize quercetin and produce butyrate (29). Interestingly, Kasai et al. reported that FP was detected more frequently in stool samples from normal subjects (0.22%) than obese subjects (0.06%) in the Japanese population (51). It is well-known that inflammation in obesity is associated with microbiota composition (52). Therefore, it is possible that FP contributes to health maintenance. In this study, IBD symptoms were suppressed by FP. In contrast, some reports have suggested the involvement of FP in intestinal disease (53–55). Dinitrobenzene sulfonic acid (DNBS)-induced ulcerative colitis mice showed a reduction in the relative abundance of FP (56). These reports suggest that among IBD, FP may promote recovery from ulcerative colitis. While FP is not thought to exhibit toxicity, further investigation of its safety is necessary (57).

In a DSS-induced acute colitis model, improvements in the disease state may involve reduced gut inflammation (58). An improvement in the percentage of body weight difference in the FP-treated group was observed on days 8, 9, and 10. No differences in food or water intake were observed in both groups. Therefore, accelerated weight regain was considered to be a consequence of the effect of FP on the host's immune system. Lenzen et al. reported that the restoration of DSS-induced colitis involves suppression of inflammatory cytokine expression, epithelial apoptosis of the colon, intestinal barrier rupture, and promotion of mucosal restoration and various transport mechanisms (59). FP may have contributed to these functions, and the link between green tea consumption and improved gut health is increasingly coming to the fore. This botanical beverage may hold benefits for people at high risk of inflammatory diseases.

## Data Availability Statement

The raw data supporting the conclusions of this article will be made available by the authors, without undue reservation.

## Ethics Statement

The animal study was reviewed and approved by the Committee for Animal Experiments of Shinshu University. Written informed consent was obtained from the owners for the participation of their animals in this study.

## Author Contributions

TO and FN conceived of and designed the experiments. AM and TO conducted the experiments. SS and TSa contributed reagents, materials, and analytical tools. AM and TSh wrote the paper. TSh supervised the work. All authors reviewed the manuscript.

## Conflict of Interest

The authors declare that the research was conducted in the absence of any commercial or financial relationships that could be construed as a potential conflict of interest.
